# Choosing Money over Drugs: The Neural Underpinnings of Difficult Choice in Chronic Cocaine Users

**DOI:** 10.1155/2014/189853

**Published:** 2014-08-14

**Authors:** Michael J. Wesley, Terry Lohrenz, Mikhail N. Koffarnus, Samuel M. McClure, Richard De La Garza, Ramiro Salas, Daisy G. Y. Thompson-Lake, Thomas F. Newton, Warren K. Bickel, P. Read Montague

**Affiliations:** ^1^Virginia Tech Carilion Research Institute, Virginia Tech, Roanoke, VA 24016, USA; ^2^Human Neuroimaging Laboratory, Virginia Tech Carilion Research Institute, 2 Riverside Circle, Roanoke, VA 24016, USA; ^3^Addiction Recovery Research Center, Virginia Tech, Roanoke, VA 24016, USA; ^4^Department of Psychology, Stanford University, Stanford, CA 94305, USA; ^5^Menninger Department of Psychiatry and Behavioral Science, Baylor College of Medicine, Houston, TX 77030, USA

## Abstract

Addiction is considered a disorder that drives individuals to choose drugs at the expense of healthier alternatives. However, chronic cocaine users (CCUs) who meet addiction criteria retain the ability to choose money in the presence of the opportunity to choose cocaine. The neural mechanisms that differentiate CCUs from non-cocaine using controls (Controls) while executing these preferred choices remain unknown. Thus, therapeutic strategies aimed at shifting preferences towards healthier alternatives remain somewhat uninformed. This study used BOLD neuroimaging to examine brain activity as fifty CCUs and Controls performed single- and cross-commodity intertemporal choice tasks for money and/or cocaine. Behavioral analyses revealed preferences for each commodity type. Imaging analyses revealed the brain activity that differentiated CCUs from Controls while choosing money over cocaine. We observed that CCUs devalued future commodities more than Controls. Choices for money as opposed to cocaine correlated with greater activity in dorsal striatum of CCUs, compared to Controls. In addition, choices for future money as opposed to immediate cocaine engaged the left dorsolateral prefrontal cortex (DLPFC) of CCUs more than Controls. These data suggest that the ability of CCUs to execute choices away from cocaine relies on activity in the dorsal striatum and left DLPFC.

## 1. Introduction

A hallmark of addiction is choosing drugs at the expense of healthier alternatives [1]. Historically, research has focused on understanding the mechanisms that give rise to the unwanted choices expressed by chronic cocaine users (CCUs) and treatment efforts have been aimed at stopping them altogether. As with many addictions, stimulant addiction is portrayed as the hijacking of natural valuation and learning signals in the brain, such as those in the dopamine-rich striatum [2]. Indeed, much scientific evidence supports the hypothesis that the neural systems that balance immediate desires with long-term gains [3–5] function suboptimally in CCUs [6–8] and the very mechanisms evolved to protect are compromised in ways that drive addicts to choose cocaine.

While this narrative organizes a wealth of experimental data, it also deemphasizes an important observation: not all decisions made by CCUs are unhealthy choices for drugs. For example, it has been demonstrated that cocaine users decrease cocaine self-administration when offered merchandise vouchers as an alternative to cocaine, with levels decreasing even further for direct money vouchers [9]. Furthermore, when cocaine users are given the choice between a 10 mg unit dose of intranasal cocaine and increasing amounts of money, choices for cocaine decrease as the amount of money offered increases [10]. The preference for money, as opposed to cocaine, in cocaine users has been referred to as a difficult but “rational” choice in the sense that individuals must forgo their abused drug for the opportunity to obtain other desired commodities [11]. A logical question becomes, “What if cocaine users are choosing money with the intent to obtain cocaine and/or other commodities later?” If so, then these individuals are planning for the future and executing an advantageous preference away from an available drug of abuse.

Hence, a tension exists between the observations that neural valuation and decision-making systems are compromised in CCUs; yet these individuals execute choice preferences away from cocaine in many domains of their life and for variable periods of time. This supports a complementary approach that seeks to understand the similarities and differences in the expression of sensible choice preferences of CCUs and non-cocaine using control participants (Controls). This will help inform therapeutic strategies that aim to increase the expression of healthier choices in CCUs. As a first step, the current study uses single- and cross-commodity intertemporal choice tasks to examine the times when individuals choose money in the presence of the opportunity to choose cocaine. Whereas single-commodity choice tasks (i.e., money now versus money later (MM) and cocaine now versus cocaine later (CC)) have been used extensively, cross-commodity tasks (i.e., money now versus cocaine later (MC) and cocaine now versus money later (CM)) have recently been introduced to examine the real-world trade-offs that CCUs experience on a daily basis [12].

In a simple depiction of the claim that reward-guided valuation and decision-making systems are compromised in CCUs, choices in favor of money, as opposed to cocaine, are likely to involve neural responses in structures other than the striatum. Hence, our working hypothesis was that choosing money instead of cocaine would not correlate with activity in the striatum but rather with other structures related to reward-guided choice. Furthermore, compensatory mechanisms would differentiate between the preference for money in CCUs and that in Controls and be observed as functional differences outside of the striatum. We demonstrate that this hypothesis is likely wrong.

## 2. Materials and Methods

### 2.1. Participants

Fifty right-handed participants were recruited from the Virginia Tech Carilion Research Center (VTCRI; Roanoke, VA, USA) or the Baylor College of Medicine (BCM; Houston, TX, USA). Twenty-five individuals were non-treatment seeking chronic cocaine users (CCUs) who met* DSM-IV* criteria for cocaine dependence and 25 individuals were control participants (Controls). Individuals were recruited via local advertisements and word of mouth and provided informed consent in accordance with the Institutional Review Board at Virginia Tech or Baylor College of Medicine. Individuals who passed inclusion criteria visited the lab on two occasions.

### 2.2. Screening and Consenting Visit

The first laboratory visit was a screening and consenting visit and the second was a scanning visit. At the beginning of each visit urinalysis tests were performed. Urinalysis kits (QuickTox Drug Screen Dip Card by Branan Medical Corporation) tested for the use of cocaine (benzoylecgonine; 300 ng/mL), amphetamine (500 ng/mL), opiates (300 ng/mL), benzodiazepines (300 ng/mL), and marijuana (delta-9-tetrahydrocannabinol; 50 ng/mL). Individuals who tested positive for any drug other than cocaine in individuals considered for the CCUs group were excluded from participation. Following initial urinalysis screening, individuals answered a series of questions related to demographic variables and were administered the Structured Clinical Interview for the Diagnostic and Statistical Manual of Mental Disorders (*DSM-IV*) [13]. Individuals who presented with a history of substance abuse for substances other than cocaine or nicotine were excluded from the study. In addition, individuals were excluded if they presented with systemic diseases of the central nervous system, head trauma, neurological disorders, or Axis-I psychiatric disorders (other than cocaine or nicotine dependence).

Addiction was operationalized based on responses to questions derived from the* DSM-IV*. Specifically, individuals were asked to reflect on their cocaine use over the past 12 months and answer a series of 7 questions that addressed tolerance; withdrawal; taking more drug than expected; failed quit attempts; time spent sequestering or thinking about cocaine; time spent away from family, hobbies, and friends; and mental health problems, respectively. The questions read as follows: (1) “Have you found that you needed to use much more cocaine to get the same effect that you did when you first started taking it?” (2) “When you reduced or stopped using cocaine, did you have withdrawal symptoms such as aches, shaking, fever, weakness, diarrhea, nausea, sweating, heart beat irregularities?” (3) “Have you found that when you used cocaine, you ended up taking more than you thought you would?" (4) “Have you tried to reduce or stop taking cocaine but failed?” (5) “On the days that you used cocaine, did you spend substantial time (>2 hours), obtaining, using, or recovering from cocaine, or thinking about using it?” (6) “Did you spend less time working, enjoying hobbies, or being with family or friends because of your cocaine use?” (7) “If cocaine caused you health or mental problems, did you still keep on using it?” Whereas individuals who responded positively (e.g., “yes”) to at least 3 of the 7 questions met criteria for cocaine dependence, individuals who responded positively to 5 or more questions were considered to be addicted to cocaine. Individuals who met criteria for cocaine addiction were included as participants in the CCUs group. Individuals who tested positive for cocaine but did not meet addiction criteria were excluded. All individuals who passed screening criteria on the first laboratory visit were invited back to the laboratory for the scanning visit. Participants in the CCUs group were asked to abstain from using cocaine starting at midnight the night before the scheduled scanning visit.

### 2.3. Scanning Visit

On the day of the scanning visit, participants arrived at the laboratory approximately two hours before the fMRI scanning session. Upon arrival individuals were administered a second urinalysis test. Since it is feasible that a positive screen for cocaine could be obtained from individuals remaining abstinent since the previous midnight (e.g., the cocaine metabolite benzoylecgonine can typically be detected in urine up to 3-4 days following use in chronic users), participants in the CCUs group who tested positive for cocaine use (100% of participants) were asked to provide the exact time since last use. They were also visually monitored for overt symptoms of acute cocaine intoxication (e.g., anxiety and agitation, excessive and/or uncontrolled motor movements, jaw clinching and/or mouth gnawing, erratic behavior, dilated pupils, etc.). Participants in the CCUs group who reported using cocaine after the previous midnight and/or were judged to be acutely intoxicated by a trained research assistant were excluded from participation (0% of participants). Participants in the Controls group were required to test negative for all drugs screened by urinalysis.

### 2.4. Choice Tasks

**Figure 1:**
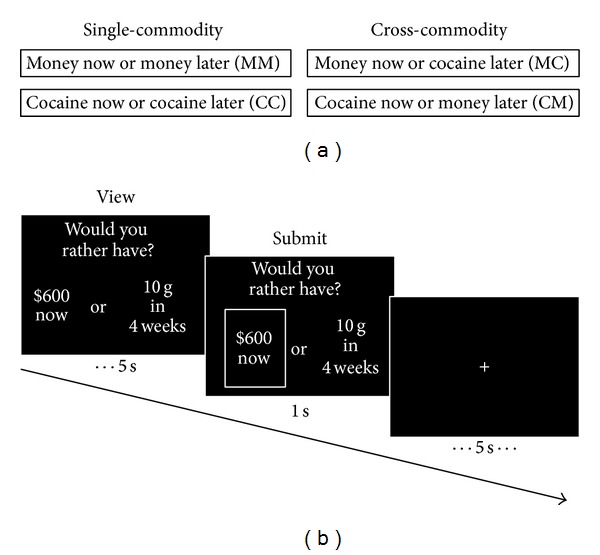
Behavioral tasks. Participants performed two single-commodity and two cross-commodity temporal decision-making tasks. (a) During single-commodity tasks individuals made choices between immediate money or delayed money (MM) and immediate cocaine or delayed cocaine (CC). In cross-commodity tasks individuals made choices between immediate money or delayed cocaine (MC) or between immediate cocaine or delayed money (CM). (b) An example trial from the MC task. A trial consisted of a viewing period lasting for a maximum of 5 s, a submission period lasting for 1 s, and a jittered fixation screen lasting for an average of 5 s. During the viewing period immediate and delayed options appeared randomly on the left or right side of the screen under the question, “Would you rather have?” Once individuals selected the immediate or delayed commodity a box appeared around their selection for 1 s. Immediate amounts were varied 6 times for each of four future time points (1, 4, 26, and 52 weeks).

The behavioral task parameters used for the current study have been described in detail elsewhere [12]. Participants performed two single-commodity and two cross-commodity discounting tasks in which they chose between hypothetical amounts of money or cocaine available immediately or after some delay (Figure 1(a)). Single-commodity tasks were tasks where participants chose between money available now or money in the future (MM) and cocaine available now or cocaine in the future (CC). Cross-commodity tasks were tasks where participants chose between money available now or cocaine in the future (MC) and cocaine available now or money in the future (CM). Prior to the task participants gave an estimate of the number of grams of cocaine that they equated with $1000. This allowed cocaine amounts to be converted to dollar amounts after the experiment. Behavioral discounting analyses were conducted using dollar amounts. Controls were asked to treat cocaine as a commodity that they had experience with.

Prior to performing behavioral tasks in the fMRI scanner, participants completed two similar single-commodity and two similar cross-commodity adjusting amount discounting tasks outside of the scanner. Indifference points, representing the amounts at which individuals are equally likely to choose a presently available commodity versus a future commodity, were calculated from the tasks performed outside the scanner and used to parameterize the scanner tasks. This was done to limit the sampling space while participants were in the scanner, maximizing the likelihood that indifference points could be calculated from data obtained from the scanner task. These procedures are described in detail in our previous study [12]. In general, participants chose between immediate and delayed amounts six times for each of seven delays (1 day, 1 week, 1 month, 6 months, 1 year, 5 years, and 25 years). The sixth choice for each delay was used as the estimated indifference point, or the value at which the participant would be indifferent between immediate and delayed options. Specifically, the initial choice at each delay was between the full large delayed amount and 50% of that amount available immediately. When participants chose one of the two options offered, the immediate amount offered in the next trial was first adjusted by ±50% of the current offer, with adjustments on subsequent trials being half of the previous adjustment. If the participant chose the immediate amount, the immediate amount decreased for the next trial; if the participant chose the delayed amount, the immediate offer increased. The indifference values obtained from 1 week, 1 month, 6 months, and 1 year were used to parameterize the offers given in the scanner tasks at 1 week, 4 weeks, 26 weeks, and 52 weeks, respectively.

An example trial from the modified behavioral task performed inside the fMRI scanner can be seen in Figure 1(b). A trial consisted of a viewing period lasting a maximum of 5 seconds. During this period, a question appeared on the top of the screen that asked, “Would you rather have?” And the immediate and future choice options appeared randomly on the left or right side of the screen below the question. Participants selected their preferred option by pressing buttons on boxes positioned in their right and left hands. As soon as a participant selected an option a box appeared around the selected option for approximately 1 second followed by a fixation screen that jittered ±1 second around and average presentation period of 5 seconds. At the end of the fixation period the next trial began. For each future time point, the amount of the immediate commodity was varied six times.

### 2.5. Functional Magnetic Resonance Imaging Data Acquisition

Images were acquired on Siemens 3T Allegra scanners either at VTCRI or at BCM. Structural T1-weighted images were first acquired (0.5 × 0.5 × 1 mm) followed by functional images with a 2 s repetition time, 25 ms echo time, and 90° flip angle in 37 interleaved slices (3.4 × 3.4 × 4 mm). Slices were hyperangulated approximately 30° from the anterior commissure-posterior commissure line in an effort to avoid orbital frontal washout. Functional data were adjusted to correct slice timing, realigned, coregistered to T1 anatomical image, normalized to Montreal Neurological Institute (MNI) coordinates, resliced (4 × 4 × 4 mm), and smoothed using an 8 mm Gaussian kernel. These steps were conducted using statistical parametric mapping (SPM08, Institute of Neurology, London, UK). Data were also high-pass-filtered at 128 s.

### 2.6. Demographic Analyses

Comparisons of parametric (i.e., age, education, and monthly income) and categorical (i.e., gender and collection site) demographic variables were conducted with independent samples *t*-tests and chi-squared tests, respectively, with significant thresholds set to *P* < 0.05.

### 2.7. Inclusion of Participants in Two Analysis Streams

**Figure 2:**
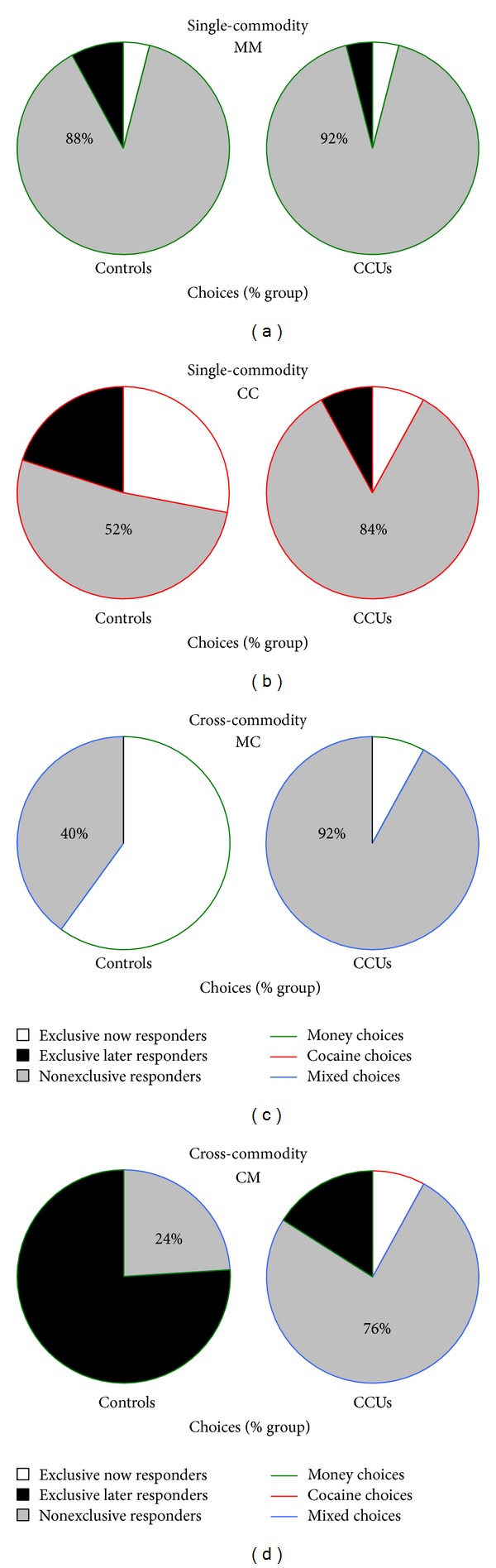
Choice behavior. Pie charts are of the proportion of each group who were exclusive (only chose immediate or delayed options) or nonexclusive (chose both immediate and delayed options) responders in each task. In single-commodity tasks individuals chose between immediate or delayed money (MM, (a)) or cocaine (CC, (b)). In cross-commodity tasks individuals chose between immediate money or delayed cocaine (MC, (c)) or between immediate cocaine or delayed money (CM, (d)). Based on choice behaviors individuals were included in two analysis streams. In the first analysis stream, nonexclusive responders (percentages displayed) were included in a temporal discounting behavioral analysis. Indifference points based on behavioral choice could not be calculated for exclusive responders, so these individuals were excluded. In the second analysis stream, all individuals (exclusive and nonexclusive responders) were included in a behavioral analysis examining all immediate and delayed choices. Exclusive responders were included according to the commodity they always chose. This analysis determined the individuals to be included in imaging analyses of immediate and delayed choices for each task.

Based on the choice behaviors exhibited by participants inside the fMRI scanner (Figure 2), individuals were further analyzed in two analysis streams. We first examined temporal discounting behavior across the four experimental tasks in nonexclusive responders, those individuals for whom indifference points could be calculated based on behavioral choices. The second stream examined behavior and brain activity for all immediate and delayed choices in each task for all individuals, allowing clear distinctions to be made between immediate and delayed money and cocaine choices. We adopted this approach to maximize the validity of the discounting analysis (e.g., only including individuals who switched choice preferences between the immediate and future options allowing calculation of an indifference point) and to maximize the number of individuals included in the imaging analysis (e.g., including individuals who did not switch between immediate and future options according to their exclusive choices).

### 2.8. Analysis Stream 1: Temporal Discounting Behavior

Only individuals who distributed choices between immediate and future options were included in the behavioral discounting analysis. First, an indifference point at each future time point, in each task, and for each individual was calculated. Indifference points were calculated as the average of the two amounts flanking a switch in choice preference between immediate and delayed options. Next, indifference points for each future time point were averaged across individuals in each group to generate indifference curves for each group in each task. Next, the area under indifference curves was calculated for each group in each task. This measure of indifference magnitude reflects the value placed on future commodities [14]. To determine if groups differed in the value placed on future commodities in single-commodity and cross-commodity tasks, 2 × 2 analyses of variance (ANOVAs) were performed on the indifference magnitudes for both the single-commodity and the cross-commodity tasks. Post hoc analyses were performed with independent samples or paired samples *t*-tests according to Bonferroni adjusted alphas.

### 2.9. Analysis Stream 2: Immediate and Delayed Choice Behavior

All individuals were included in this behavioral analysis according to their immediate and delayed choices. For each individual, the percentage of choices allocated to immediate and delayed options was calculated in each task. Percentages were then averaged by group and task type. Independent samples or paired samples *t*-tests were used to examine if groups differed in the proportion of immediate (now) and delayed (later) choices in each task.

### 2.10. Analysis Stream 2: Immediate and Delayed Choice Imaging

To determine if Controls and CCUs differed in functional brain activity while viewing and submitting choices for immediate and future options, general linear models were created for each individual. For each individual, a multiple linear regression was performed with delta functions set to the onset times for the viewing and submission times for all immediate and delayed responses. Viewing times were separated retroactively according to immediate and delayed choice submissions. This was done to examine activity that preceded the behavioral expression of choice associated with immediate and delayed options. Activity for each stimulus type was convolved with a canonical hemodynamic response function. For each individual, beta maps of activity associated with viewing and submitting both immediate and delayed options were directly compared between groups for each task. To account for potential variance associated with undesired variables and consistent with previous methods [15], the parametric variables of age in years, education in years, and number of cigarettes smoked per day were entered as nuisance variables in all random-effects analyses. Comparisons were thresholded with a voxelwise* P* value of 0.005 (familywise error corrected at the cluster level).

## 3. Results

### 3.1. Demographics

The average (±SD) age of Controls was 39.9 (±22.2) years and did not significantly differ from 34.7 (±20.9) years in CCUs, *t*(48) = 0.369, *P* > 0.05. The average (±SD) length of education in Controls was 14 (±6.7) years and did not differ from 14 (±6.3) years in CCUs, *t*(48) = 0.678, *P* > 0.05. The average (±SD) number of cigarettes smoked by Controls was 2.1 (±3.6) per day and did not differ from 4.5 (±4.2) cigarettes smoked by CCUs, *t*(48) = 0.18, *P* > 0.05. The average (±SD) monthly income of Controls was $888.29 (±619.26) and did not differ from $857.65 (±782.37) in CCUs, *t*(48) = 0.127, *P* > 0.05. The distribution of Controls and CCUs collected at the two experimental locations did not differ from expected values (BCM: Controls = 14, CCUs = 20; VTCRI: Controls = 11, CCUs = 5), *χ* ^2^ (1, *n* = 50) = 3.309, *P* > 0.05. The number of males and females in each experimental group significantly differed from expected values (Controls: males = 13, females = 12, CCUs: males = 20, females = 5), *χ* ^2^(1, *n* = 50) = 4.367, *P* < 0.05. Within- and between-group analyses of both behavioral and brain imaging data, however, yielded no significant differences related to gender.

Participants in the CCUs group reported an average (±SD) of 14.58 (±4.5) years of experience using cocaine and reported using cocaine for 24.14 (±7) days out of the previous month. Fourteen participants in the CCUs group reported smoking crack cocaine as their primary method of delivery. All participants in the CCUs group met criteria for cocaine addiction according to questions derived from the* DSM-IV* (see Materials and Methods). Ten members of the Controls group reported having previous experience with marijuana use, while twenty-five members of the CCUs group reported having previous experience with marijuana use. All participants reported having previous experience with alcohol use. At the time of the study, however, all participants tested negative for illicit drug use other than cocaine in CCUs. Furthermore, participants did not reach criteria for substance dependence, with the exception of cocaine and nicotine dependence in CCUs and nicotine dependence in Controls. Nicotine use (cigarettes smoked per day) was treated as a nuisance variable in all imaging analyses (see Section 2).

### 3.2. Two Analysis Streams

Based on choice behaviors on the discounting tasks performed in the fMRI scanner individuals were included in two analysis streams (Figure 2). Only nonexclusive responders were included in the first analysis stream of temporal discounting behavior. For the single-commodity tasks, this resulted in 92% of CCUs (*n* = 23) and 88% of Controls (*n* = 22) included in the MM groups. The CC groups consisted of 84% of CCUs (*n* = 21) and 52% of Controls (*n* = 13). In the cross-commodity tasks, 92% of CCUs (*n* = 23) and 40% of Controls (*n* = 10) were included in the MC groups. The CM groups consisted of 76% of CCUs (*n* = 19) and 24% of Controls (*n* = 06). In the second analysis stream, all individuals (exclusive and nonexclusive responders) were included according to their choices for immediate and delayed commodities. The numbers of participants included in behavioral and corresponding imaging analyses of the second analysis stream can be observed in Tables 1 and 2.

### 3.3. Analysis Stream 1: Temporal Discounting Behavior

**Figure 3:**
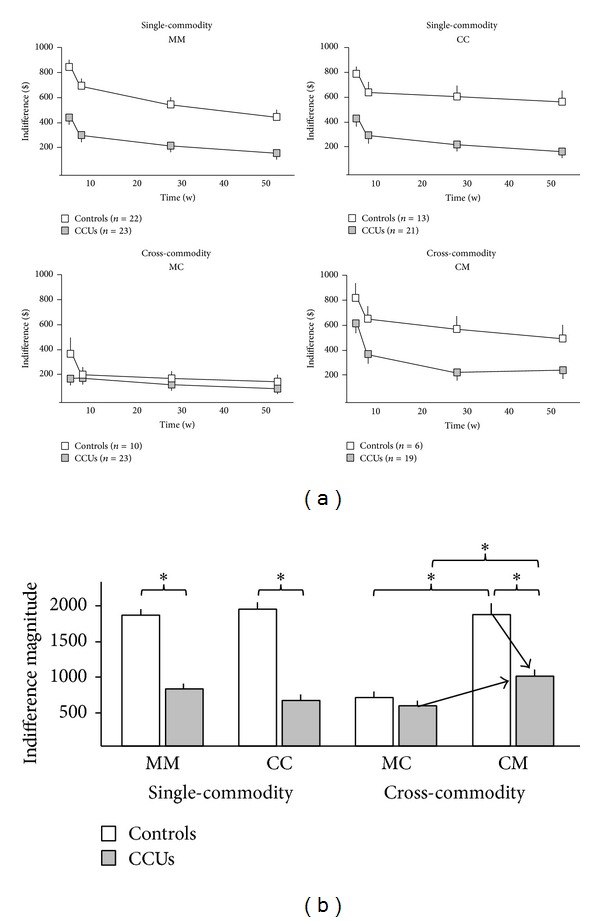
Temporal discounting behavior. (a) Indifference amounts (mean ± SEM) at each of four future time points (1, 4, 26, and 52 weeks) in nonexclusive responders in each group for single-commodity and cross-commodity tasks. In single-commodity tasks individuals chose between immediate or delayed money (MM) or cocaine (CC). In cross-commodity tasks individuals chose between immediate money or delayed cocaine (MC) or between immediate cocaine or delayed money (CM). (b) Comparison of indifference magnitudes, measured by area under the indifference curves in each task. Larger magnitudes reflect more value placed on the future. Conversely, smaller values reflect less value placed on the future. CCUs devalued future money and cocaine more than Controls during single-commodity tasks. During cross-commodity tasks, arrows point out that while CCUs devalue delayed money more than Controls, they valued delayed money significantly more than delayed cocaine. *P < 0.01.

In the first stream, average indifference magnitudes (±SEM) revealed the value each group placed on future commodities. These results are shown in Figure 3. In the single-commodity tasks a main effect of group was observed when examining indifference magnitudes (*F*(1, 75) = 39.871, *P* < 0.001). Post hoc analysis demonstrated that the indifference magnitudes (mean ± SEM) in the MM task were significantly less in CCUs (837 ± 146), compared to Controls (1896 ± 152) (*t*(43) = 5.170, *P* < 0.001). Similarly, indifference magnitudes in the CC task were significantly less in CCUs (612 ± 132), compared to Controls (1921 ± 232) (*t*(32) = 3.783, *P* = 0.001). Within-group analysis of single-commodity tasks revealed that indifference magnitudes did not differ between MM and CC within Controls (*t*(31) = 0.92, n.s). Similarly, indifference magnitudes did not differ between MM and CC within CCUs (*t*(31) = 0.92, n.s.).

In the cross-commodity tasks main effects were observed for group (*F*(1, 54) = 5.98, *P* = 0.018) and task type (*F*(1, 54) = 20.18, *P* < 0.001). Post hoc comparisons revealed that CCUs (457 ± 124) and Controls (640 ± 203) did not differ in indifference magnitudes during the MC task (*t*(31) = 0.791, n.s.). However, during the CM task, indifference magnitudes in CCUs (942 ± 129) were significantly less than Controls (1860 ± 281) (*t*(23) = 2.304, *P* < 0.031). Within-group analysis of cross-commodity task types revealed that indifference magnitudes for Controls were significantly greater in CM, compared to MC (*t*(14) = 3.59, *P* < 0.003). Similarly, indifference magnitudes in CCUs were significantly greater in CM, compared to MC (*t*(40) = 2.85, *P* = 0.007), suggesting that both Controls and CCUs value future money more than future cocaine.

### 3.4. Analysis Stream 2: Immediate and Delayed Choice Behavior

**Figure 4:**
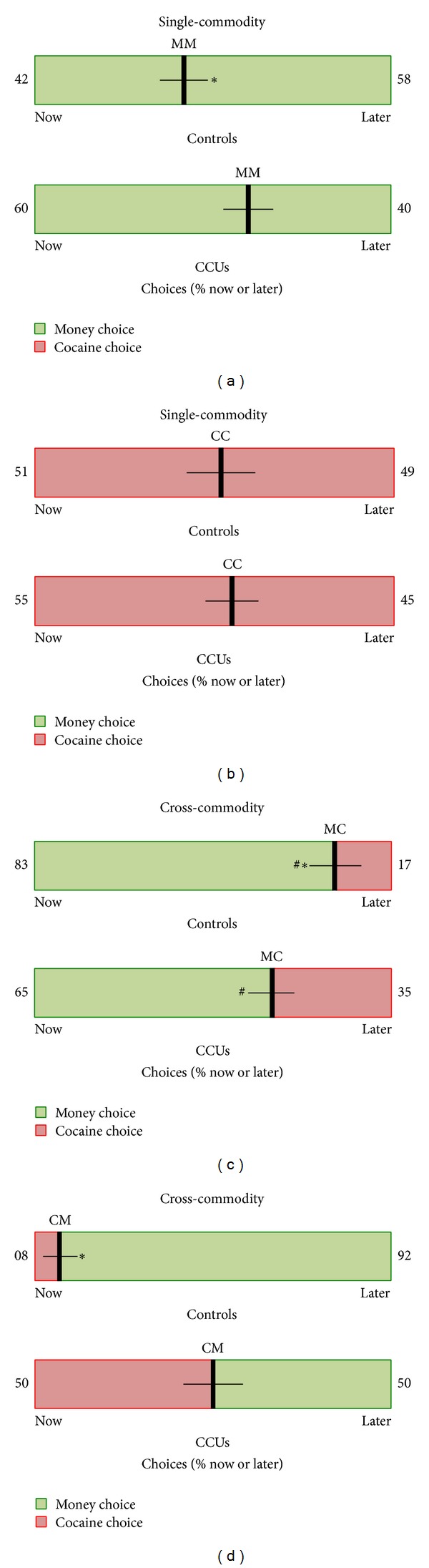
Money and cocaine choices. Data are the averaged (±SEM) percentages of immediate (now) and future (later) choices in controls (Controls) chronic cocaine users (CCUs). Single-commodity choice involved choosing between immediate or future money (MM) or cocaine (CC), and cross-commodity choice involved choosing between immediate money and future cocaine (MC) or between immediate cocaine and future money (CM). Money and cocaine choices are green and red, respectively. CCUs made fewer choices for future money, compared to Controls (MM and CM). In cross-commodity tasks, CCUs chose cocaine more than Controls. Regardless, a large portion of the choices were for money (MC = 65% and CM = 50%). Incentivizing the future with money resulted in a 15% increase in future choices in CCUs away from the immediate cocaine option (MC = 35% versus CM = 50%). ∗Difference between groups, within task, *P* < 0.01; ^#^difference, within group, between cross-commodity tasks, *P* < 0.05.

The group percentages (mean ± SEM) for all immediate and delayed choices in each group are displayed in Figure 4. During the MM task, the percentage of delayed money choices made by CCUs (40 ± 4.9) was significantly less than Controls (58 ± 4.7) (*t*(48) = 2.5, *P* = 0.016). No difference was observed between groups during the CC task (*t*(48) = 0.62, n.s.). During the cross-commodity MC task, CCUs made significantly more delayed cocaine responses (35 ± 4.3) than Controls (17 ± 5.3) (*t*(48) = 2.7, *P* = 0.01). During the cross-commodity CM task, CCUs made significantly less delayed money responses (50 ± 6.4) than Controls (92 ± 3.8) (*t*(40) = 5.5, *P* < 0.001). Examining the proportion of choices within groups during the cross-commodity tasks revealed that behavioral choices followed the money commodity in both groups. This can be seen as the proportion of delayed responses between MC and CM significantly differed, in favor of the money option, in both Controls (*t*(24) = 9.0, *P* < .001) and CCUs (*t*(24) = 2.4, *P* = 0.03).

### 3.5. Analysis Stream 2: Functional Imaging Results

General linear models targeted brain activity while submitting immediate (now) or delayed (later) choices and retrogradely isolated activity while viewing options that became now or later choices. Very few differences between groups were observed in the single-commodity tasks (Table 1). In the MM task, viewing choice options for what became money now choices resulted in greater precuneus activity in CCUs, compared to Controls. On the other hand, viewing what became money later choices resulted in greater inferior frontal gyrus and inferior temporal lobe activity in Controls, compared to CCUs. In the CC task, submitting cocaine now choices produced greater temporal lobe and postcentral gyral activity in Controls, compared to CCUs. Cocaine later choices resulted in greater medial prefrontal activity in Controls, compared to CCUs.

**Figure 5:**
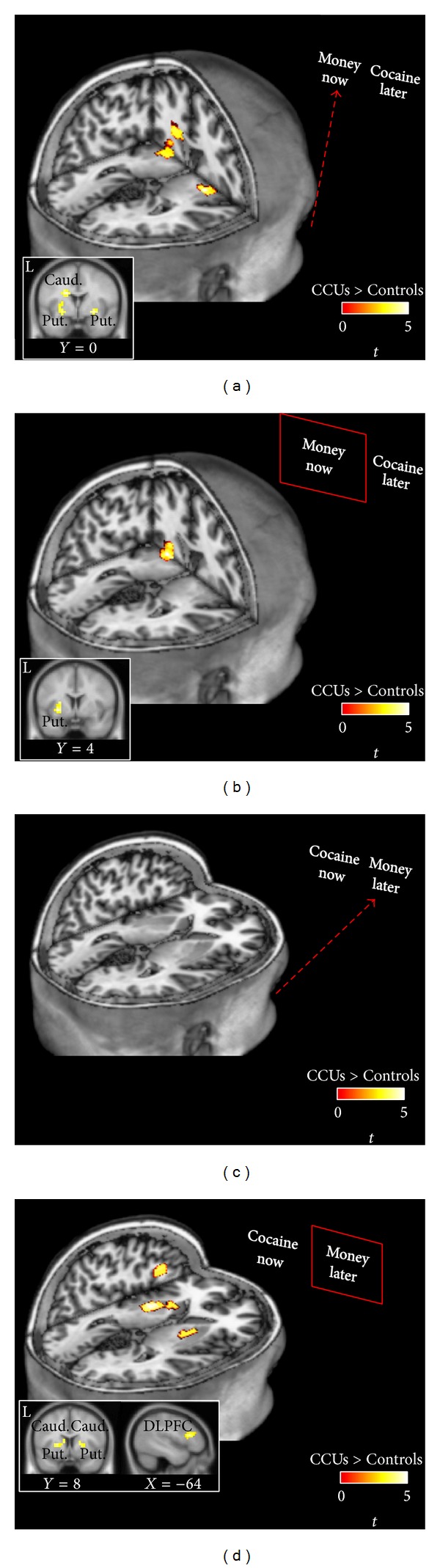
Chronic cocaine users choosing money instead of cocaine. Data are functional clusters where chronic cocaine users (CCUs) had greater responses than controls (Controls) for money choices in cross-commodity tasks. Choices were between immediate money and future cocaine (MC: (a), (b)) or between immediate cocaine and future money (CM: (c), (d)). Each individual's immediate (now) and future (later) choices were used to isolate activity while viewing (dashed red line) what became money choices (a), (c) and while submitting money choices (b), (d). Activity in the striatal putamen, caudate, and globus pallidus was greater in CCUs while viewing what became money now choices (a) and while executing money later choices (d). In addition, activity in the left dorsolateral prefrontal cortex was greater for money later choices.

Imaging results during cross-commodity tasks are presented in Figure 5 and Table 2. During cross-commodity tasks, signals in the striatum and left lateral prefrontal cortex differentiated CCUs from Controls. While viewing options for what became money now choices, CCUs had significantly greater activity in bilateral putamen, globus pallidus, and the left caudate (Figure 5(a)). Activity in the left putamen persisted as CCUs executed money now choices (Figure 5(b)). Groups did not differ while viewing options that became money later choices (Figure 5(c)). However, spatially coincident striatal responses, as well as a response in the left dorsal lateral prefrontal cortex, emerged when CCUs executed money later choices (Figure 5(d)).

## 4. Discussion

We used single- and cross-commodity choice tasks and fMRI to examine the neurofunctional events that distinguish non-cocaine choice preferences made by chronic cocaine users (CCUs) from those of controls (Controls). Consistent with previous behavioral studies, we observed that CCUs devalued the future more than Controls, as measured by diminished indifference magnitudes in the single-commodity context. In the cross-commodity context, both CCUs and Controls executed several choices for money when cocaine was the available alternative. Compared to Controls, choices for immediate money as opposed to future cocaine were associated with greater activity in the dorsal striatum of CCUs. In addition to greater dorsal striatal activity, choices for future money as opposed to immediate cocaine, were associated with greater left dorsal lateral prefrontal cortex (DLPFC) activity in CCUs, compared to Controls.

Our behavioral analyses of the single-commodity tasks (i.e., money now versus money later (MM) and cocaine now versus cocaine later (CC)) revealed that CCUs devalued future money significantly more than Controls. This was observed in the first analysis as significantly smaller indifference magnitudes during the MM task in CCUs, compared to Controls, and was confirmed in the second analysis where CCUs chose future money 18% less often than Controls (40% versus 58%). This is consistent with previous single-commodity studies demonstrating that, relative to non-cocaine using individuals, cocaine addicts devalue the future [7].

In the cross-commodity context (i.e., money now versus cocaine later (MC) and cocaine now versus money later (CM)), we found further evidence that CCUs devalue future money, relative to Controls. CCUs had significantly smaller indifference magnitudes during the CM task, compared to Controls, and chose future money 42% less often than Controls (50% versus 92%). Perhaps not surprisingly, when given the choice between money and cocaine, the majority of choices in Controls were for money (83% in MC and 92% in CM). Interestingly, a large portion of responses in CCUs was also for money (65% in MC and 50% in CM). This allowed direct comparisons to be made between groups for money choices in the cross-commodity context. Also of note, in the cross-commodity context offering money in the future resulted in a 15% increase in future choices made by CCUs. This is a clear demonstration that a desired commodity engenders the ability to shift choices in CCUs away from their drug of abuse towards a nondrug alternative for the future and is consistent with previous studies demonstrating the ability of cocaine users to forgo cocaine for money [9, 10].

In addition to demonstrating that CCUs retain the ability to shift choices away from cocaine, these behavioral results are interesting for another reason. Using a single dataset, we present data with seemingly conflicting interpretations. On one hand, we show that CCUs devalue the future, a finding so prevalent in various disease states that it is considered a transdisease process [16]. On the other hand, we confirm previous behavioral results that CCUs will forgo available drug when an alternative commodity is incentivized appropriately. This highlights the complexity in understanding real-world trade-offs in CCUs and should motivate future studies to tease apart such complexities. In the current study, given that both Controls and CCUs executed choice preferences for money, as opposed to cocaine, we were able to probe the neurofunctional signals that differentiated CCUs from Controls.

Imaging analysis of the single-commodity choices revealed that during the MM task, inferior frontal and temporal lobe activity was greater in Controls, compared to CCUs, while viewing future money choices. On the other hand, CCUs exhibited greater precuneus activity while viewing immediate money choices, relative to Controls. During the CC task, Controls displayed greater temporal lobe and postcentral gyrus activity while choosing immediate cocaine, compared to CCUs, and greater medial prefrontal activity (mPFC) for cocaine later choices. These results suggest that Controls place more value on future commodities, compared to CCUs, regardless of the commodity type and this is associated with increased executive function-related activity in Controls, as opposed to CCUs.

We found that when choosing money as opposed to cocaine, CCUs relied on greater activity than Controls in areas known to be involved in normal valuation and decision-making [4, 17–20]. Namely, activity in the caudate and putamen of the dorsal striatum was greater as CCUs viewed and executed money choices. In addition to the role it plays in the expression of habitual behaviors, the midbrain dopamine system, including the dorsal striatum, has been studied extensively for its specific role in the picoeconomics and neuroeconomics of gambling disorders [21]. Furthermore, it is hypothesized that disruptions in the dorsal striatum underlie the cocaine seeking behaviors that accompany chronic cocaine exposure in both rodent [22, 23] and primate [24, 25] models of addiction. In addition, CCUs displayed greater activity in the left DLPFC when choosing future money over immediate cocaine, compared to Controls. The left DLPFC is well known for its executive functioning role in on-going decision-making, and our results are consistent with a recent meta-analysis demonstrating that this area is where temporal discounting processes related to the future and working memory processes related to the recent past overlap in the brain [26]. Therefore, it is feasible that greater activity in the left DLPFC of CCUs is required when executing a difficult choice preference for a future commodity while forgoing immediate cocaine. These results are consistent with a model where CCUs exhibit brain function above the levels of Controls when executing a choice preference for money as opposed to cocaine.

The results from the current study are novel and relevant in light of previous studies that have suggested inhibiting cue-induced striatal dopamine increases as a therapeutic strategy for cocaine addiction [27]. This suggestion logically follows the observation that dopamine levels in the dorsal striatum are positively associated with drug craving [27] and drug craving is known to contribute to relapse. This approach is also supported by data from experiments in primates demonstrating that the transition from acute to chronic cocaine self-administration results in a progressive shift in dopamine receptor expression and glucose utilization from the ventral to the dorsal striatum [24, 25]. The shift from acute cocaine effects in the ventral striatum, impairing goal-directed choice, to chronic cocaine effects in the dorsal striatum, impairing habitual choice, has been hypothesized to underlie many addiction-related dysfunctions in cocaine abusing humans (for review see [28]). Our results suggest, however, that simply inhibiting activity in the dorsal striatum, while being potentially beneficial for treating some of the negative aspects of addiction, may inadvertently compromise the ability of cocaine addicts to execute choice preferences away from available cocaine. The current data suggests that therapeutic approaches should consider the potential confound of compromising real-world relevant, cross-commodity choices. We contend that successful strategies will benefit from more detailed understanding of the brain activity associated with nondrug choices in CCUs.

Activity in the left DLPFC was greater in CCUs, compared to Controls, while executing choices for future money as opposed to immediate cocaine. The lateral PFC is known to be active during decision-making and is particularly active when considering the costs and benefits of alternatives [29]. Recently, a transcranial magnetic stimulation (TMS) study found that disrupting the left, but not the right, DLPFC increased choices of immediate rewards over delayed rewards, providing a causal link between activity in the left DLPFC and self-control mechanisms of future choice [30]. Our data are consistent with these results and the competing neurobehavioral decision systems hypothesis, which suggests that the evolutionarily young executive system (including the DLPFC) works in concert with the older limbic system for optimal decision-making [31, 32]. When these systems are functioning suboptimally decision-making becomes impaired. For example, hypofrontal activity is also associated with compromised executive abilities in various disease states, including schizophrenia [33] and major depression [34]. Consistent with rodent studies demonstrating that repeated self-administration of cocaine decreases basal levels of PFC activity [35], methamphetamine-abusing populations have been observed to have prefrontal hypoactivity during future choice tasks, compared to Controls [8]. Our data suggests that, compared to Controls, increased DLPFC is needed in conjunction with increased dorsal striatal activity for CCUs to execute choices for alternative future commodities when immediate cocaine is also an available commodity. Our results suggest that the left DLPFC is a unique therapeutic target for shifting choices in CCUs towards healthier alternatives for the future.

### 4.1. Limitations

Several variables should be considered when interpreting the current study. Although we relied on strict methodological standards for screening out current illicit drug use and dependence on substances other than cocaine (our key experimental variable) and nicotine (not different between groups and controlled for in imaging analyses), results may be influenced by extraneous variables not under our explicit experimental control (e.g., gender, age, education, cocaine use patterns, and poly drug, and nontarget drug use). While analysis of these variables, with the exception of gender, revealed no significant difference between groups, it is possible that these variables contributed in some way to the observed results. Additional imaging analyses performed according to gender yielded no significant differences. It should be noted, however, that dividing groups by gender reduced group sample sizes to numbers often deemed underpowered for neuroimaging studies. Future studies will explicitly test gender differences associated with nondrug choices in chronic cocaine users.

The commodities used in the current study were hypothetical in nature. As such, they are less likely to evoke visceral responses understood to influence decision-making processes [36]. This could represent an additional source of variance in the current study. It is worth noting, however, that fictive and real money gains and losses have been demonstrated to produce similar behaviors and activate similar brain networks during intertemporal choice tasks, including activity in the striatum and lateral prefrontal cortices [37]. Nonetheless, it is plausible that due to their inexperience with cocaine, Controls need not exert self-control comparable to that of the CCUs when choosing money over cocaine. It is a possibility that CCUs, on the other hand, experience willpower or planning proficiency (even in the hypothetical case) not present in Controls. To inform this possibility, and in search of additional neural signals associated with choosing money, we performed additional random-effects analyses in each experimental group comparing single- and cross-commodity tasks (i.e., MM versus CM and MM versus MC). These analyses, however, did not yield significant results. This may reflect a lack of statistical power due to the current design and the limited choice behaviors exhibited by participants. Finally, in the current study, addiction was operationalized as affirmative responses to five or more (out of seven)* DSM-IV* criteria used to establish cocaine dependence (which requires a minimum of three of seven affirmative responses; see Materials and Methods). Therefore, the definition of addiction in the current study is somewhat arbitrary and our results may be generalizable only to a subgroup of cocaine dependent individuals who meet a high number of dependence criteria as established by the* DSM-IV*. In light of these potential limitations, we encourage readers to treat these data as preliminary. Notwithstanding, we hold that the significant results obtained from between-group comparisons of money choices in the cross-commodity context, when cocaine is the nonchosen commodity, are novel, valid, and informative. These data should be considered in future accounts of therapies aimed at shifting choice behaviors in chronic cocaine users.

## 5. Conclusion

In conclusion, the cross-commodity context can be used as an experimental paradigm to isolate times when CCUs forgo their drug of abuse for a preferred alternative. Our hypothesis that money choices in CCUs would not rely on brain activity in the striatum was rejected. Updating this hypothesis, we propose that the difficult choice to forgo cocaine in CCUs relies on hyperactivity in the dorsal striatum and DLPFC, known valuation and decision-making brain areas. Specifically, hyperactivity in the dorsal striatum is needed for immediate and future choice preferences away from cocaine, and coincident hyperactivity in the left DLPFC is needed for future choice preferences away from cocaine. Activity in these areas represents therapeutic targets for shifting choices in CCUs away from cocaine options.

## Figures and Tables

**Table 1:** Areas of significant BOLD signal differences between controls (Controls) and chronic cocaine users (CCUs) while viewing and submitting choices during single-commodity blocks of money now versus money later (MM) and cocaine now versus cocaine later (CC).

Conditions	H	Area	Coordinates			*k*_*E*_	*t*-value
			*x*	*y*	*z*		
MM							
View							
Money now							
Controls (23) > CCUs (24)	**n/a**						
CCUs (24) > Controls (23)	R	Precuneus	6	−52	26	10	3.10
Money later							
Controls (24) > CCUs (24)	L	IFG	−54	24	18	12	3.16
	R	Inferior temporal	54	−44	−10	17	3.57
CCUs (24) > Controls (24)	**n/a**						
Submit							
Money now							
Controls (23) > CCUs (24)	**n/a**						
CCUs (24) > Controls (23)	**n/a**						
Money later							
Controls (23) > CCUs (24)	**n/a**						
CCUs (24) > Controls (23)	**n/a**						

CC							
View							
Cocaine now							
Controls (20) > CCUs (23)	**n/a**						
CCUs (23) > Controls (20)	**n/a**						
Cocaine later							
Controls (18) > CCUs (23)	**n/a**						
CCUs (23) > Controls (18)	**n/a**						
Submit							
Cocaine now							
Controls (20) > CCUs (23)	R	Temporal lobe	38	0	−22	10	3.37
	L	Postcentral gyrus	−34	−28	50	11	3.03
CCUs (23) > Controls (20)	**n/a**						
Cocaine later							
Controls (18) > CCUs (23)	L	mPFC	6	48	30	10	3.30
CCUs (23) > Controls (18)	**n/a**						

H: hemisphere. Listed areas correspond to location of the maximum voxel of activation with the activity cluster. Coordinates are listed in standard MNI space.

**Table 2:** Areas of significant BOLD signal differences between controls (Controls) and chronic cocaine users (CCUs) while viewing and submitting choices during cross-commodity blocks of money now versus cocaine later (MC) and cocaine now versus money later (CM).

Conditions	H	Area	Coordinates			*k*_*E*_	*t*-value
			*x*	*y*	*z*		
MC							
View							
Money now							
Controls (25) > CCUs (25)	L	Primary visual	−22	−88	−6	178	7.88
	R	Primary visual	30	−84	−6	97	7.01
CCUs (25) > Controls (25)	L	Caudate	−18	0	26	15	3.93
	R	Putamen	26	−4	−2	15	3.88
Cocaine later							
Controls (10) > CCUs (23)	**n/a**						
CCUs (23) > Controls (10)	**n/a**						
Submit							
Money now							
Controls (25) > CCUs (25)	**n/a**						
CCUs (25) > Controls (25)	L	Putamen	−30	4	−2	13	3.89
Cocaine later							
Controls (10) > CCUs (23)	R	Angular gyrus	50	−60	34	12	3.62
	L	Mid. temporal gyrus	−54	−64	10	13	3.53
	R	Sup. temporal gyrus	46	−40	14	11	3.43
CCUs (23) > Controls (10)	L	vmPFC	−18	36	−10	11	3.74

CM							
View							
Cocaine now							
Controls (6) > CCUs (21)	**n/a**						
CCUs (21) > Controls (6)	**n/a**						
Money later							
Controls (25) > CCUs (23)	**n/a**						
CCUs (23) > Controls (25)	**n/a**						
Submit							
Cocaine now							
Controls (6) > CCUs (21)	**n/a**						
CCUs (21) > Controls (6)	**n/a**						
Money later							
Controls (25) > CCUs (23)	**n/a**						
CCUs (23) > Controls (25)	L	Putamen	−26	4	6	17	3.76
	R	Putamen	18	4	6	15	3.24
	L	DLPFC	−46	24	18	12	3.28
	R	Sup. parietal lobe	34	−72	46	11	3.63

H: hemisphere. Listed areas correspond to location of the maximum voxel of activation with the activity cluster. Sup.: superior. Mid.: middle. DLPFC: dorsolateral prefrontal cortex. Coordinates are listed in standard MNI space.
